# In Situ Monitoring of MicroRNA Replacement Efficacy and Accurate Imaging‐Guided Cancer Therapy through Light‐Up Inter‐Polyelectrolyte Nanocomplexes

**DOI:** 10.1002/advs.201700542

**Published:** 2018-01-19

**Authors:** Xiongwei Deng, Zhaoxia Yin, Jianqing Lu, Xianlei Li, Leihou Shao, Caiyan Zhao, Yishu Yang, Qin Hu, Yan Wu, Wang Sheng

**Affiliations:** ^1^ College of Life Science and Bioengineering Beijing University of Technology No. 100 Pingleyuan Beijing 100124 P. R. China; ^2^ National Center for Nanoscience and Technology No. 11 Beiyitiao Zhongguancun Beijing 100190 P. R. China

**Keywords:** glutathione, imaging‐guided therapy, in situ monitoring, inter‐polyelectrolyte nanocomplexes, microRNA‐34a

## Abstract

Replacement of downregulated tumor‐suppressive microRNA (Ts‐miRNA) is recognized as an alternative approach for tumor gene therapy. However, in situ monitoring of miRNA replacement efficacy in a real‐time manner via noninvasive imaging is continually challenging. Here, glutathione (GSH)‐activated light‐up peptide‐polysaccharide‐inter‐polyelectrolyte nanocomplexes are established through self‐assembly of carboxymethyl dextran with disulfide‐bridged (“S—S”) oligoarginine peptide (S‐Arg_4_), in which microRNA‐34a (miR‐34a) and indocyanine green (ICG) are simultaneously embedded and the nanocomplexes are subsequently stabilized by intermolecular cross‐linking. Upon confinement within the robust nanocomplexes, the near‐infrared fluorescence (NIRF) of ICG is considerably quenched (“off”) due to the aggregation‐caused quenching effect. However, after intracellular delivery, the disulfide bond in S‐Arg_4_ can be cleaved by intracellular GSH, which leads to the dissociation of nanocomplexes and triggers the simultaneous release of miR‐34a and ICG. The NIRF of ICG is concomitantly activated through dequenching of the aggregated ICG. Very interestingly, a good correlation between time‐dependent increase in NIRF intensity and miR‐34a replacement efficacy is found in nanocomplexes‐treated tumor cells and tumor tissues through either intratumoral or intravenous injections. Systemic nanocomplexes‐mediated miR‐34a replacement significantly suppresses the growth of HepG‐2‐ and MDA‐MB‐231‐derived tumor xenografts, and provides a pronounced survival benefit in these animal models.

## 1 Introduction

MicroRNA (miRNA) represents a class of endogenous, small, and highly conserved nonprotein‐coding RNAs that acts as post‐transcriptional gene regulators in almost all physiological process.^1^ It has been proposed that aberrant expression of miRNA was strongly associated with numerous biological processes as well as pathological processes.^2^ Dysregulation of miRNA has been discovered in almost all types of human cancers, which may function as either tumor suppressors or oncogenes.^3^ More importantly, rectification of miRNA abnormality by miRNA replacement has emerged as one of novel strategies for alternative miRNA‐based tumor therapy.^4^ However, transportation of miRNA to the tumor sites is still dramatically hindered by multistage biological barriers including enzymatic degradation, clearance in blood, and poor cellular uptake.^5^ A variety of established nanostructures have been successfully developed so far for miRNA delivery, including liposomes/lipids, polymeric micelles, inorganic/metal nanomaterials, and DNA/RNA assembly nanostructures, which have drastically facilitated the clinical translation of miRNA replacement therapy.^6^, ^7^, ^8^, ^9^

In situ monitoring of cargo delivery in a real‐time manner is of great importance in personalized cancer therapy. Over the past years, emerging studies have shown that synergistic integration of imaging modalities and therapeutics into one single theranostic platform could provide information regarding the biodistribution, release and assessment of the therapeutic responses, which was defined as imaging‐guided therapy.^10^, ^11^, ^12^ However, it should be mentioned that conventional “always on” theranostics are defective because fluorescence signals are emitted regardless of their real location in the focal zone and as a result displaying poor signal‐to‐noise ratio (S/N ratio) or even “false positive” results.^13^ To address this issue, reversibly activatable light‐up nanoprobes are of great need to be developed to emit signals from “off‐state” to “on‐state” in the site of interest to improve S/N ratio.^14^, ^15^, ^16^, ^17^, ^18^ Moreover, such platforms provide great potential to be used for real‐time monitoring of the transportation and release of “drug/gene” in tumor cells and tumor tissues.^19^ Indocyanine green (ICG) is an U.S. FDA‐approved near‐infrared fluorescence (NIRF) that has been widely used in imaging and photodynamic therapy community, due to its sharp and intense absorption and fluorescence in the red to NIR region.^20^, ^21^ However, the confinement of ICG showed a notorious phenomenon with weakened or self‐quenched fluorescence intensity due to π–π stacking and other nonradiative pathways, named as aggregated‐caused quenching (ACQ).^22^ It has been demonstrated that incorporation of ICG monomers into well‐defined nanostructures may result in ACQ due to multistaged intermolecular and/or intramolecular interactions in a small space.^23^ In this account, controlled conversion of the self‐quenched ICG in rationally designed nanostructures to monomers is of great interest to be developed to perform fluorescence signal transition from “off‐state” to “on‐state” for accurate imaging‐guided tumor therapy and real‐time monitoring capability.

Nanosized supramolecular inter‐polyelectrolyte complexes (NS‐IPECs) present novel opportunities for self‐assembled macromolecules and have been receiving intensive attention in drug/gene encapsulation, delivery, and controlled release.^24^, ^25^ Indeed, delivery strategy based on NS‐IPECs presents a facile, mild, and green approach, without involving harsh process and organic solvents, which is beneficial to preserve the drug/gene activity. Various types of building blocks have been devised so far for NS‐IPECs preparation, such as natural and synthetic polymers, biomimetic macromolecules, and inorganic/metal ions, which showed low toxicity, high translational values, tunable functionality, and ease of production.^26^, ^27^, ^28^ In particular, positively charged oligoarginines have raised special interest, which possess several advantages including cell penetrating, nucleic acids binding capability, synthetic versatility, accessibility for tagging/labeling, and the easy design of various stimuli‐responsive sequences.^29^, ^30^, ^31^ At present, various stimuli‐responsive “smart” nanostructures have been developed according to the heterogeneity of extra‐ and intratumoral microenvironments, such as low pH, altered redox potential, hypoxia, and dysregulated enzymes, which allow spatially controlled release of the therapeutics only in the sites of interest to improve therapeutic efficiency.^32^, ^33^, ^34^, ^35^ For instance, the concentration of glutathione (GSH) is remarkably higher in tumor cells (10 × 10^−3^ m) compared to that in the blood plasma whose concentration is about 2 × 10^−6^ to 10 × 10^−6^ m.^36^ Disulfide bonds (“S—S”) are stable in an oxidizing extracellular environment and are rapidly reduced in an intracellular environment due to the thiol‐disulfide exchange reaction with high concentration of GSH.^37^ This transformation has thus encouraged the design and development of disulfide‐bridged “smart” nanoparticles for GSH‐responsive delivery.

**Scheme 1:**
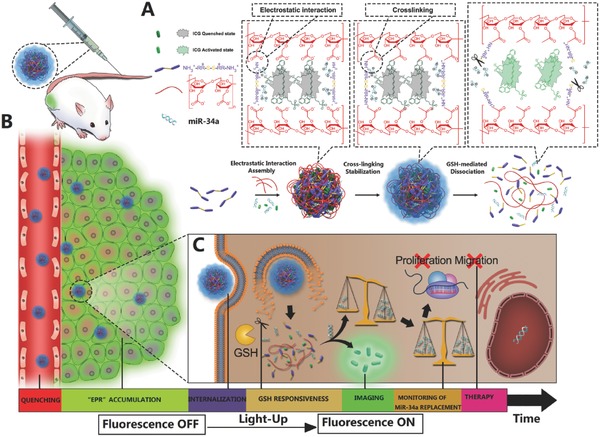
Schematic illustration of the design, construction, and application of light‐up CMINs for monitoring of miR‐34a replacement efficacy and accurate imaging‐guided cancer therapy. A) Chemical structures of the components and preparation procedures of CMINs. At optimal ratio, S‐Arg_4_ and CMD could self‐assemble into spherical inter‐polyelectrolyte nanocomplexes and simultaneously embed miR‐34a and ICG. Further cross‐linking endows high stability of nanocomplexes and prevents miR‐34a degradation. ICG tends to form aggregate state upon embedding within CMINs, resulting in self‐quenching effect and fluorescence “off” state. B) Schematic of the accumulation of CMINs in tumor sites via EPR effect (passive targeting). C) Schematic of GSH‐mediated dissociation of CMINs and the release of ICG and miR‐34a upon intracellular uptake of nanocomplexes, which led to the activation of ICG fluorescence from “off‐state” to “on‐state” for monitoring of miR‐34a replacement efficacy and accurate imaging‐guided miR‐34a‐based tumor therapy.

In this study, peptide‐polysaccharide inter‐polyelectrolyte nanocomplexes were designed and generated through self‐assembly of polysaccharide of carboxymethyl dextran (CMD) and disulfide‐linked oligoarmine, which can be specially dissociated by intracellular GSH, and have a great potential to act as GSH‐responsive platform. ICG and miR‐34a were simultaneously coembedded in the nanocomplexes and subsequently stabilized by intermolecular cross‐linking (referred to as CMINs), which could better protect the embedded miR‐34a and ICG and insure their release especially when dissociation of nanocomplexes happens. In our design, ICG was used as the NIRF probe which tends to aggregate while entrapped within the nanocomplexes and exhibits no fluorescence (“off‐state”) due to self‐quenching effect (**Scheme** **1**A). ICG may be simultaneously released with miR‐34a in tumor cells upon intracellular uptake and dissociation of nanocomplexes by intracellular GSH, in which the NIRF of ICG can be reversibly recovered (“on‐state”). MiR‐34a has been identified as one of the most potent and well‐defined Ts‐miRNA in a variety of tumors. Since the miR‐34a release with the dissociation of CMINs in cancer cells is accompanied by a simultaneously activating NIRF of ICG, the increase in the NIRF signal readout could be utilized as an indirect signal to monitor intracellular miR‐34a replacement efficacy in situ. CMINs‐mediated replacement of miR‐34a may thus inhibit tumor progression through downregulating the expression of downstream targets both in vitro and in vivo. The as‐prepared light‐up nanocomplexes provide therefore multiple potential advantages, including passive accumulation at tumor sites due to the enhanced permeability and retention (EPR) effect, activatable tumor imaging pattern with high specificity and reduced background signal, in situ monitoring of miR‐34a release and replacement efficacy in vitro/vivo, effective miR‐34a replacement therapy without obvious side effects, which hold great promise for accurate imaging‐guided miR‐34a‐based theranostic application (Scheme 1B,C).

## 2 Results and Discussion

### 2.1 Construction and Characterization of Peptide‐Polysaccharide‐Inter‐Polyelectrolyte Nanocomplexes

Peptide composed of four arginines was synthesized and intramolecularly bridged with a disulfide bond (“S—S”) (Arg–Arg–S–S–Arg–Arg, abbreviated as S‐Arg_4_), using solid‐phase peptide synthesis method, which acquired a high density of positive charge with redox‐sensitive disulfide bonds. S‐Arg_4_ peptides were then purified by high performance liquid chromatography and characterized by liquid chromatography‐mass spectrometry analyses (Figures S1 and S2, Supporting Information). It has been reported that such oligoarginines containing cationic arginine rich regions are able to condense short nucleic acid via electrostatic interactions.^38^ CMD was selected as the polyanionic polyelectrolyte in our formulation based on the following considerations: it is a polysaccharide approved for clinical use with excellent biocompability and widely utilized as a surface‐coating component similar to polyethyleneglycol (PEG) in nanomedicine to enhance blood circulation of nanoparticles.^39^ CMD was synthesized according to the method reported previously^40^ (Scheme S1, Supporting Information) and characterized by Fourier transform infrared spectroscopy (FT‐IR) analysis (Figure S3, Supporting Information).

**Figure 1:**
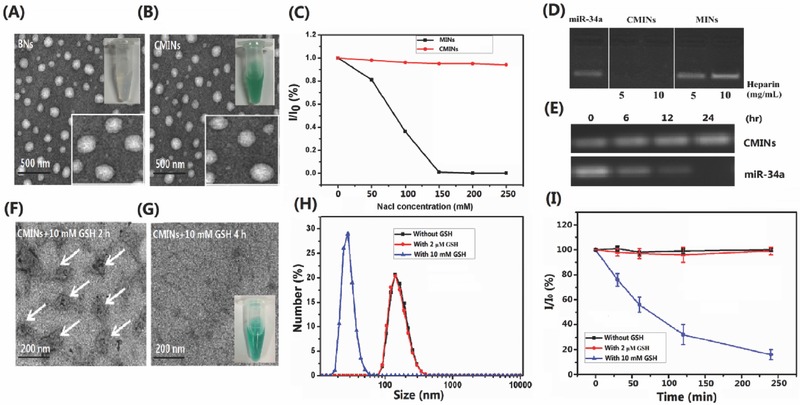
Physical–chemical properties of the constructed inter‐polyelectrolyte nanocomplexes. TEM images of A) BNs and B) CMINs. Inset images in (A) and (B) were the photographs of BNs and CMINs dispersed in water. C) Kinetic stability of MINs and CMINs was assessed by monitoring the scattering light intensity by DLS analysis under conditions of various concentrations of NaCl. D) Stability of MINs and CMINs after incubation with different concentrations of heparin. E) MiR‐34a stability in serum environment at extended time points. TEM images of CMINs treated with 10 × 10^−3^ m GSH for F) 2 h and G) 4 h. The images demonstrate the gradual dissociation of CMINs upon GSH treatment. H) DLS analysis of CMINs dispersed in PBS of pH 7.4 without GSH and with 2 × 10^−6^ m and 10 × 10^−3^ m GSH. I) Dissociation kinetics of CMINs in PBS buffer (7.4) with 2 × 10^−6^ m GSH and 10 × 10^−3^ m GSH or without GSH detected by relative scattering light intensity. Data were shown as average of triplicate measurements with standard error bars.

Blank peptide‐polysaccharide‐inter‐polyelectrolyte nanocomplexes (referred to as BNs) were self‐assembled by mixing S‐Arg_4_ and CMD dissolved in aqueous solution slowly via electrostatic interactions between the positively charged tertiary amine arising from S‐Arg_4_ and the negative charges from the ionizable carboxyl group of the CMD. Different mass ratios of S‐Arg_4_ and CMD were mixed to optimize construction condition by measuring the sizes, polydispersity index (PDI), zeta potentials, and product yields. We found that the mass ratio of S‐Arg_4_ to CMD of 1.1:1 was suitable for nanocomplexes formation with the optimal hydrodynamic size (<200 nm), PDI (<0.2), and relative high product yield (>70%) and this construction condition was chosen in our following experiments. Dynamic light scattering (DLS) measurements revealed that the average diameter of BNs was ≈182 nm with unimodal size distribution (PDI = 0.172) and the zeta potential was negatively charged (−17 mV) at this formulation (Figure S4A–D, Supporting Information). Moreover, transmission electron microscopy (TEM) imaging revealed that BNs exhibited a spherical morphology in shape with an average diameter of ≈100 nm (**Figure** **1**A). It should be noted that the sizes of BNs acquired by TEM measurements leads to smaller measured diameter relative to average hydrodynamic diameter via DLS measurements, which may be caused by dehydration and partial collapse of BNs corona in dry state. MiR‐34a and ICG were further embedded into the peptide‐polysaccharide‐inter‐polyelectrolyte nanocomplexes (denoted as MINs). ICG is able to non‐covalently interact with a variety of proteins or peptides via various physical mechanisms, including electrostatic, hydrophobic, and hydrogen bonding interactions.^41^, ^42^ MiR‐34a can be embedded into the inter‐polyelectrolyte nanocomplexes due to the negatively charged property of nucleic acid.^43^ The embedding efficiencies (EE) and loading content (LC) of ICG and miR‐34a were then optimized by mixing various mass ratios of the four components. As shown in Table S1 (Supporting Information), the mass ratio of S‐Arg_4_, CMD, MiR‐34a to ICG of 1.1:1:0.05:0.05 is suitable to obtain optimal EE and LC. The EE of miR‐34a and ICG in this formulation were 75.2% and 70.2% and the LC of miR‐34a and ICG were 2.55% and 2.45%, respectively. The average size of MINs slightly increased to about 192 nm and the zeta potential decreased slightly to about −18.2 mV upon embedding of miR‐34a and ICG (Figure S5, Supporting Information). 1‐ethyl‐3‐(3‐dimethylaminopropyl) carbodiimide hydrochloride (EDC) was furthermore used as a cross‐linking agent to covalently link S‐Arg_4_ and CMD through amine–carboxy coupling in a mild reaction condition to increase the stability (referred to as CMINs).^44^ DLS measurements showed a slight increase in the size of CMINs (≈212 nm) compared with MINs (≈192 nm) (Figure S6, Supporting Information). TEM imaging also showed that the as‐prepared CMINs were well‐dispersed and spherical‐shaped and morphologically similar to BNs (Figure 1B), which indicated that embedding of miR‐34a and ICG and cross‐linking had negligible influence on the morphology of the nanocomplexes. As shown in Figure 1C, the resultant cross‐linked CMINs were stable when exposed to various ionic strength compared with uncross‐linked MINs. Moreover, electrophoresis analysis showed that no extraction of miR‐34a was observed from CMINs upon incubation with 5 and 10 mg mL^−1^ of heparin. By contrast, miR‐34a extraction was clearly found in MINs treated with the above concentrations of heparin (Figure 1D), indicating that cross‐linking efficiently improved the stability of CMINs. Furthermore, the analysis of miR‐34a stability in serum demonstrated that CMINs could protect miR‐34a from endogenous nuclease digestion without obvious degradation for up to 24 h compared with free miR‐34a in the presence of serum (Figure 1E). Moreover, CMINs remained colloidal stable in various solutions and maintained their sizes after one week of storage at room temperature (Figure S7, Supporting Information). Next, bovine serum albumin (BSA) was used as a model protein to evaluate the potential protein adsorption ability of CMINs. As shown in Figure S8 (Supporting Information), CMINs showed minimal protein adsorption after 2 and 4 h of incubation, indicating their ability to avoid nonspecific interaction with serum components, which is consistent with previous reports.^40^ The above data suggested that CMINs were stable in various solutions and remarkably prevented miR‐34a degradation.

### 2.2 GSH‐Responsive Properties of CMINs

The GSH‐responsive properties of CMINs were subsequently investigated by exposing CMINs to two concentrations of GSH buffers of 2 × 10^−6^ m and 10 × 10^−3^ m over predetermined time, which mimic the GSH concentration in human plasma and intracellular environment, respectively.^36^ As shown in Figure 1F,G, TEM images illustrated that CMINs were partially disassembled and switched into an amorphous state after 2 h of incubation with 10 × 10^−3^ m GSH and completely disassembled after 4 h of incubation. By contrast, CMINs maintained their initial uniform morphology and size after incubation with 2 × 10^−6^ m GSH for both 2 and 4 h (Figure S9, Supporting Information). Additionally, the results of DLS measurements were in accordance with the TEM observations (Figure 1H), exhibiting a decrease in the sizes of CMINs upon incubation with 10 × 10^−3^ m GSH. The dissociation kinetics of CMINs was thereafter quantitatively evaluated by monitoring the changes of scattering light intensity (SLI). As shown in Figure 1I, the dissociation of CMINs was evidenced by the decreasing SLI from 100% to ≈10% during 4 h of incubation with 10 × 10^−3^ m GSH, while no significant changes in the SLI of CMINs were found in the absence of GSH and in the presence of 2 × 10^−6^ m GSH. These results revealed that CMINs were gradually dissociated by responding to high concentration of GSH indentified in intracellular compartments and not responsive to low level of GSH found in serum.

**Figure 2:**
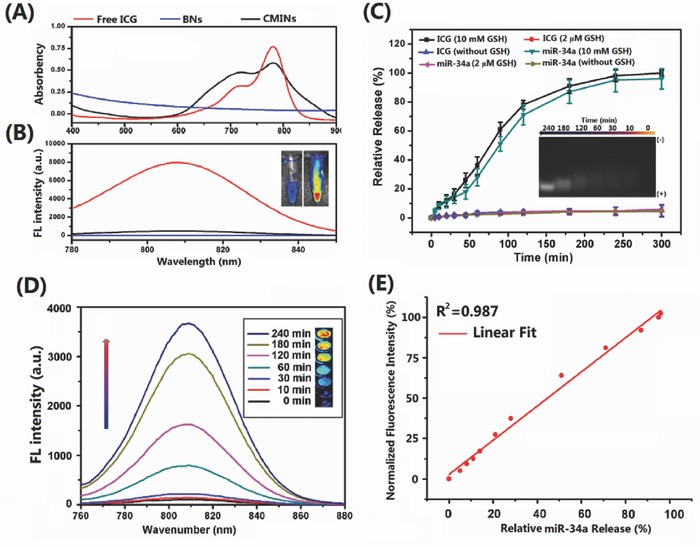
A) UV/vis spectra and B) fluorescence spectra of ICG solution, BNs and CMINs. The inset is the corresponding photograph of ICG solution (left) and CMINs (right) obtained by CRi ex/in vivo imaging system. C) ICG and miR‐34a release profiles. Data were demonstrated as average of triplicate measurements with standard error bars. The inset showed miR‐34a release visualized by gel electrophoresis. D) Time‐dependent fluorescence spectra of CMINs in the presence of 10 × 10^−3^ m GSH. The inset was the NIRF image sections of CMINs incubated with 10 × 10^−3^ m GSH in 96‐well microplate at different time intervals. E) Correlation of the normalized fluorescence intensity of ICG and miR‐34a release upon treatment with 10 × 10^−3^ m GSH at different time intervals.

The optical properties of the CMINs were then investigated with UV–vis spectrophotometer, fluorescence emission spectra, and CRi ex/in vivo imaging system. As shown in **Figure** **2**A, ultraviolet‐visible (UV‐vis) absorption spectra analysis demonstrated that, compared to free ICG, which had strong absorption peak at 780 nm and a relative weak absorption at 720 nm, respectively, absorption curve were remarkably altered upon its embedding into CMINs. The data implied that embedding of ICG into CMINs greatly affected the original structure of ICG monomer. In addition, compared with free ICG, no significant fluorescence was detected in CMINs as well as in BNs while excited at 780 nm compared with free ICG (Figure 2B), which implied the ACQ effect of embedded ICG in CMINs. CMINs‐embedding‐mediated quenching property of ICG was also visualized with a CRi ex/in vivo imaging system, which also demonstrated a significant NIRF quenching of ICG upon CMINs embedding (inset in Figure 2B).

### 2.3 Monitoring of MiR‐34a Release by Assessing the “Off–On” NIRF

The release of miR‐34a and ICG from CMINs was subsequently investigated. Cy‐3‐labeled miR‐34a was used for quantitative evaluation of miR‐34a release. As shown in Figure 2C, treatment with 10 × 10^−3^ m GSH led to a rapid and cumulative release of miR‐34a and ICG, which reached a maximum of about 92% and 96% within 4 h, respectively. In sharp contrast, no significant release of miR‐34a and ICG was detected in CMINs without GSH treatment or treated by 2 × 10^−6^ m GSH, indicating an effective responsiveness of CMINs to intracellular concentration of GSH. GSH‐triggered release of miR‐34a from CMINs was also visualized by agarose electrophoresis analysis. Gradual release of miR‐34a from CMINs was detected upon 10 × 10^−3^ m GSH treatment within 4 h (inset in Figure 2C). Significantly, a good correlation between the release ratios of miR‐34a and ICG was observed (Figure S10, Supporting Information), indicating a concomitant release of miR‐34a and ICG upon 10 × 10^−3^ m GSH treatment. In addition, similar absorption curves were observed in free ICG and 10 × 10^−3^ m GSH‐treated CMINs for 4 h (Figure S11, Supporting Information), suggesting that ICG can be efficiently released from CMINs and underwent a conversion from aggregate state to monomers state upon treatment with 10 × 10^−3^ m GSH. Moreover, NIRF intensity of ICG increased following the extension of GSH‐incubation time within 4 h and reached 35‐fold higher than original NIRF intensity at 4 h postincubation (Figure 2D). In addition, NIRF images of GSH‐treated CMINs were visualized at extended time points by using the CRi ex/in vivo imaging system upon treatment by 10 × 10^−3^ m GSH. Gradually increased NIRF signals were observed following the extension of incubation time incubation with 10 × 10^−3^ m GSH (inset in Figure 2D), indicating that deaggregated ICG molecules were progressively released from the dissembled CMINs and switched from “off‐state” to “on‐state.” The above results revealed that CMINs exhibited NIRF light‐up property upon treatment with 10 × 10^−3^ m GSH.

We thereafter investigated the potential of established CMINs to monitor miR‐34a release by quantitatively assessing the NIRF intensity of released ICG upon treatment with 10 × 10^−3^ m GSH. For quantitative visualization, we plotted normalized fluorescence intensity (NFI = [*F*–*F*_OFF_]/[*F*_ON_–*F*_OFF_]) as an index of fluorescence recovery, where *F* is the fluorescence intensity of the CMINs at different time points incubated with 10 × 10^−3^ m GSH, and *F*_ON_ and *F*_OFF_ represent the maximal and minimal fluorescence intensities of CMINs at the “on‐state” and “off‐state,” respectively. The relationship of the ratios of miR‐34a release with the NFI values was subsequently was analyzed upon treatment of CMINs with 10 × 10^−3^ m GSH. The data showed that the NFI value increased linearly with the released level of miR‐34a with a linear coefficient of 0.987 (Figure 2E), indicating a good linear relationship between the recovered fluorescence intensity of ICG and the level of released miR‐34a. The obtained results suggested that the GSH‐responsive light‐up CMINs were capable of monitoring miR‐34a release by recovered ICG fluorescence in a 10 × 10^−3^ m GSH environment.

### 2.4 Cellular Uptake, Fluorescence Imaging, and Monitoring Intracellular MiR‐34a Replacement Efficacy by CMINs Treatment

**Figure 3:**
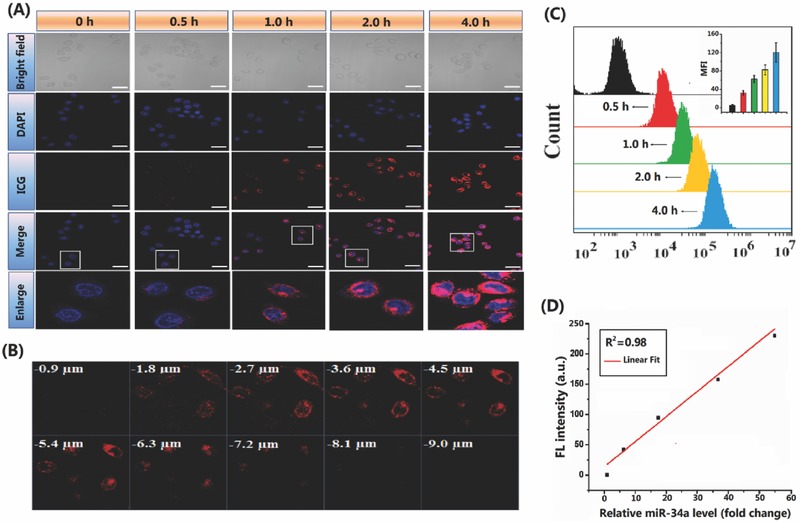
NIRF imaging and monitoring of miR‐34a replacement efficacy using CMINs in HepG‐2 cancer cells. A) Confocal images of HepG‐2 cells treated by CMINs for 0.5, 1, 2, and 4 h at 37 °C. Cells were counterstained with DAPI (nuclei, blue). Scale bars in all images represent 10 µm. B) Z‐stack images were taken within a total depth of 9 µm, with 0.9 µm depth of each slice. C) Flow cytometry analysis of cells treated with CMINs for 0.5, 1, 2, and 4 h. D) Response curve between relative fluorescence intensity of ICG and intracellular miR‐34a level in HepG‐2 cells measured by qRT‐PCR assay after CMINs treatment (100 × 10^−9^ m). E) Data were represented by mean ± SD (*n* = 3, **p < 0.01).

The cytotoxicity of the nanocomplexes was primarily assessed using HepG‐2 cell as a cell model by cell counting kit‐8 (CCK‐8) assay. Scramble miRNA was embedded into CMINs (referred to as Scr‐CMINs) for cytotoxicity assays. The results showed that Scr‐CMINs did not cause any detectable cytotoxicity in all tested cases (Figure S12, Supporting Information), indicating a good biocompatibility of the established nanocomplexes. Time‐course intracellular NIRF imaging was thereafter investigated by confocal laser scanning microscopy. Cultured HepG‐2 cells were treated by CMINs for 0.5, 1, 2, and 4 h, respectively. As shown in **Figure** **3**A, fluorescence signal of ICG remarkably increased along with treatment times, indicating an efficient intracellular uptake of CMINs, effective release and recovered fluorescence of ICG in HepG‐2 cells. Z‐stack confocal images of cells were recorded along the *z*‐axis after 4 h of incubation and clear distance‐dependent fluorescence signal was observed. The red intensity increased with increasing cell depth until 5.4 µm and then decreased (Figure 3B). Furthermore, time‐course intracellular fluorescence of ICG in HepG‐2 cells was also measured by flow cytometric analysis (FACS). The fluorescence intensity increased with the elongation of incubation time, which is consistent with above results (Figure 3C). The replacement efficacy of miR‐34a using CMINs was therefore quantitatively measured by qRT‐PCR method. Interestingly, we found a good correlation between the relative fluorescence intensity of ICG and the miR‐34a level in cytoplasm (Figure 3D), suggesting that the intracellular released amount of miR‐34a can be monitored by measuring relative fluorescence intensity of ICG in living cells in a noninvasive imaging manner. Taken together, the above results illustrated that the GSH‐responsive CMINs were able to mediate fluorescence transition of ICG from “off‐state” to “on‐state” in tumor cells, which is suitable for quantitatively monitoring intracellular miR‐34a release and replacement.

### 2.5 In Vitro Anticancer Efficacies of MiR‐34a Replacement by CMINs Treatment

**Figure 4:**
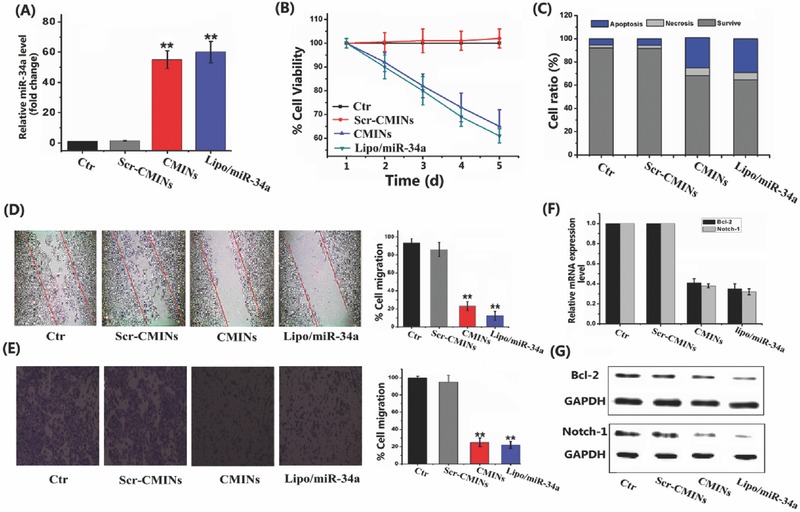
Biological effects of CMINs‐mediated replacement of miR‐34a in HepG‐2 cancer cells. A) Relative miR‐34a level in cancer cells at 48 h post‐treatment with Scr‐CMINs, CMINs and lipo/miR‐34a. B) Cell proliferation was quantitatively measured by using a cell counting kit‐8 (CCK‐8). C) Cell apoptosis was analyzed by flow cytometry. Apoptotic evaluation was illustrated by the percentage of apoptotic cell number relative to total cell number. D) Wound healing and E) transwell assays were conducted to evaluate cell migration. Migration ratio was quantitatively shown by normalized gray values. The expression of Bcl‐2 and Notch‐1 in HepG‐2 cells 48 h after treatment with different formulations by F) RT‐PCR and G) western blot analysis. Data were represented by mean ± SD (*n* = 3, Student's *t*‐test, **p < 0.01).

The biological effects of CMINs‐mediated intracellular replacement of miR‐34a were thus investigated and compared with miR‐34a‐loaded commercial lipofectamine (denoted as lipo/miR‐34a). No‐treated cells and Scr‐CMINs‐treated cells were used as controls to measure endogenous miR‐34a level. As shown in **Figure** **4**A, low level of miR‐34a was detected in no‐treated cells as well as Scr‐CMINs‐treated cells. By contrast, high level of miR‐34a was found in both CMINs‐ and lipo/miR‐34a‐treated cells. The upregulation of miR‐34a expression by CMINs was about 50‐fold higher than control groups and with a similar efficiency to lipo/miR‐34a. The proliferation, apoptosis, and migration of tumor cells were subsequently investigated upon treatment of different formulations. The present results demonstrated that, compared to the controls, CMINs‐ and lipo/miR‐34a‐meiated intracellular replacement of miR‐34a were able to inhibit tumor cell proliferation (Figure 4B), enhance tumor cell apoptosis (Figure 4C) as well as cell migration, which was investigated by both wound healing assay (Figure 4D) and transwell assays (Figure 4E). For wound healing and transwell assays, we normalized with the total cell number at the endpoint of these experiments. As shown in Figure S13 (Supporting Information), the total number of CMINs‐treated cells remained up to 90% compared with the control groups. These results demonstrated that the significant reduction of number of migrative cells was not due to the decreased cell growth. It has previously been reported that Bcl‐2 and Notch‐1 genes were both well identified as downstream targets of miR‐34a.^45^ The expression of these two genes was quantitatively examined by quantitative reverse transcription‐polymerase chain reaction (qRT‐PCR) and western‐blot, respectively. Downregulation of Bcl‐2 and Notch‐1 was found both in CMINs‐ and lipo/miR‐34a‐treated HepG‐2 cells compared to the controls at both transcriptional mRNA level (Figure 4F) and translational protein level (Figure 4G), suggesting that miR‐34a delivery provided by CMINs for the efficient replacement of miR‐34a level and play a significant suppressive role in tumor cells through inhibiting the expression of downstream targets such as Bcl‐2 and Notch‐1. Furthermore, the effects of serum concentrations (0% to 75%) on the expression of Bcl‐2 and Notch‐1 after treatment with CMINs and lipo/miR‐34a were evaluated, respectively. The downregulation efficiencies of the expression of Bcl‐2 and Notch‐1 were reduced following the increase of serum concentrations in lipo/miR‐34a‐treated cells. Conversely, no significant alterations were found on the expression of both genes in CMINs‐treated cells (Figure S14, Supporting Information), indicating a negligible effects of serum on intracellular uptakes of CMINs, which is favorable for in vivo application of CMINs. Hemocompatibility of CMINs was further evaluated by hemolytic assays. As shown in Figure S15 (Supporting Information), CMINs induced negligible hemolysis in all cases, indicating a good hemocompatibility for the established nanocomplexes. In addition, very weak fluorescence signals were detected in CMINs compared to 10 × 10^−3^ m GSH‐treated CMINs, which were both dispersed in mice blood 24 h postincubation. The data suggested that CMINs were stable in mice blood with very weak ICG fluorescence, which can be activated by the decomposition of CMINs upon GSH treatment (Figure S16, Supporting Information).

### 2.6 In Vivo Activated NIRF‐Bioimaging and Monitoring MiR‐34a Replacement Efficacy

**Figure 5:**
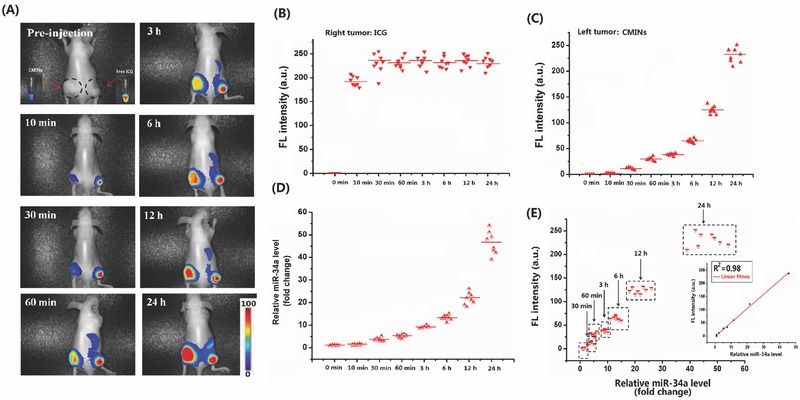
NIRF imaging‐guided monitoring of miR‐34a replacement efficacy in nude mice bearing subcutaneous tumors by local injection. A) In vivo time‐course NIRF imaging of two HepG‐2 tumors injected with CMINs (left tumor) and ICG solution (right tumor). The black‐dashed circles indicated the tumor sites. Relative fluorescence intensity of right tumor injected with B) ICG solution and C) left tumor injected with CMINs at different time intervals. D) Relative miR‐34a level in the left tumors at different time intervals. E) Correlation between relative fluorescence intensity of ICG with miR‐34a level in tumors after treatment with CMINs. The fluorescence intensity was linearly correlated with miR‐34a level with R^2^ = 0.98. Data represent the average of eight independent experiments (*n* = 8 mice per group).

Subsequently, NIRF imaging‐guided monitoring of miR‐34a replacement efficacy was evaluated in tumor‐bearing nude mice by local intratumoral injection of CMINs. HepG‐2 cells were inoculated subcutaneously in both left and right flank to establish two xenograft tumors. 50 µL of CMINs dispersed in phosphate‐buffered saline (PBS) (100 µL of CMINs contain 3 µg of ICG and 3.3 µg of miR‐34a) and free ICG solution (2 µg of ICG) were injected concurrently into the left and right tumors, respectively. In vivo NIRF images were then captured at indicated time points upon injection. As shown in **Figure** **5**A, NIRF signal in the left side tumor was obviously lower than that of right side tumor within 6 h postinjection. However, NIRF intensity increased significantly over time in the left side tumor, which was similar to that of right side tumor injected by free ICG at 24 h postinjection (Figure 5B,C). The obtained data implied an efficient internalization of CMINs by tumor cells, which led to gradually intracellular release of ICG and reversely activated ICG NIRF signals. Intratumoral time‐course replacement efficacy of miR‐34a was further analyzed by qRT‐PCR in tumors extracted at indicated time intervals (Figure 5D). Gradually increased miR‐34a level was found in our analysis, which showed a good correlation with the increased NIRF intensity in xenograft tumors (Figure 5H). These findings matched well with the in vitro studies and confirmed the feasibility of our NIRF light‐up CMINs to monitor miR‐34a replacement efficacy in vivo by local injection.

**Figure 6:**
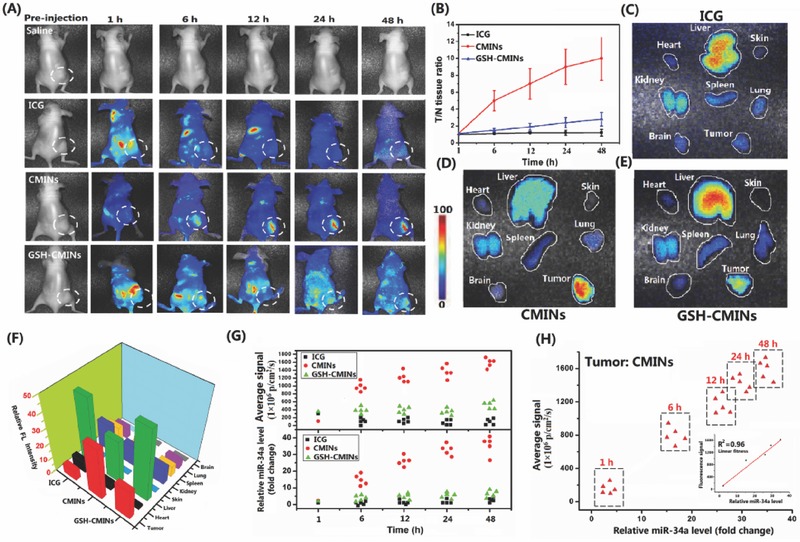
A) Time course of NIRF images of subcutaneous HepG‐2 xenograft tumors in nude mice after intravenous administration of ICG solution, CMINs, and GSH‐CMINs. The white‐dashed circles indicated the tumor sites. B) NIRF intensity ratio between tumors and normal tissues (T/N ratio) as a function of time. Ex vivo fluorescence images of major organs and tumor tissue from HepG‐2 xenograft mice sacrificed at 48 h postinjection of C) ICG, D) CMINs, and E) GSH‐CMINs. F) Quantitative analysis of NIRF intensity from tumors and major organs. G) Quantitative analysis of relative NIRF intensity of ICG and miR‐34a level in xenograft tumors at indicated time points after intravenous administration of different formulations. Data represent mean ± SD (*n* = 5, Student's *t*‐test, **p < 0.01). H) Correlation between relative fluorescence intensity of ICG with miR‐34a level in tumors after treatment with CMINs. The fluorescence intensity was linearly correlated with miR‐34a level with R^2^ = 0.96.

Furthermore, in vivo NIRF imaging and monitoring of miR‐34a delivery and replacement were investigated in xenograft mice model via systemic tail‐vein injection of CMINs. The mice were imaged at 1, 6, 12, 24, and 48 h postinjection of saline, ICG solution and 10 × 10^−3^ m GSH‐treated CMINs for 2 h (GSH‐CMINs), respectively. As shown in **Figure** **6**A, the NIRF signals of ICG were predominantly detected in liver and abdomen in mice received injection of ICG solution and GSH‐CMINs within 12 h postinjection in which no significant NIRF signals could be detected in tumor sites over 48 h postinjection. In sharp contrast, the NIRF signals were specifically dominated in tumor sites in CMINs‐treated mice after 6 h post‐treatment, in which the NIRF signal intensity increased over time within 48 h of treatment (Figure 6A). The obtained results suggested that, compared with free ICG and GSH‐CMINs without tumor targeting ability, ICG can be specifically delivered into tumor tissues by CMINs and released in tumor cells, which allowed to be detected by NIRF imaging. It should be mentioned that, compared to the controls in which NIRF signals could be obviously observed in liver and abdomen, no significant NIRF signals in the whole body were detected in CMINs‐treated mice other than tumor tissues up to 48 h of observation, further demonstrating that NIRF of ICG in CMINs was constantly quenched during the circulation in blood and activated after intracellular uptake by tumor cells. The ratio of the NIRF intensity in tumor tissues and in normal tissues was quantitatively measured and shown in Figure 6B. We found that NIRF intensity of ICG remarkably increased over time in xenograft tumors in CMINs‐treated mice and reached tenfold higher than that in normal tissues. In comparison, NIRF intensity only slightly augmented over time in xenograft tumors in mice received injection of GSH‐CMINs compared to free ICG‐injected mice in which no increase of NIRF intensity in tumor tissues was observed over time compared with normal tissues. Furthermore, the tumor and representative organs were dissected after treatment with different formulations for 48 h and then probed by ex vivo fluorescence imaging analysis. Consistently, it was clearly identified that intense NIRF signals were observed in the tumor of mice received tail‐vein injection of CMINs whereas moderate to low levels of NIRF were present in the kidney, heart, spleen, lung, brain, and skin. However, the NIRF signals were observed dominantly in livers rather than tumors in both free ICG‐ and GSH‐CMINs‐treated mice, which are consistent with the above data (Figure 6C–F). The frozen tumor sections of ex vivo tumor tissue at 48 h postadminstration were observed by fluorescence microscopy (Figure S17, Supporting Information). For CMINs‐treated mice, the tumor slice showed strong fluorescence, while there was almost no fluorescence observed for the other groups. The obtained results demonstrated that systemically injected with CMINs could accumulate at tumor sites effectively and present activatable tumor imaging pattern with high specificity and reduced background signal.

To verify the possibility of monitoring miR‐34a replacement efficacy through tail‐vein injection of CMINs, NIRF intensity and miR‐34a level in xenograft tumors were also quantitatively compared. Free ICG and GSH‐CMINs‐treated mice were used as controls. Tumor tissues were isolated at indicated time points upon injection and the miR‐34a levels in tumors were quantified by qRT‐PCR method. Interestingly, we found that miR‐34a level was significantly increased in tumor tissues over time in comparison with a moderate increase of miR‐34a level in GSH‐CMINs‐treated mice. However, no significant augmentation of miR‐34a level was detected in free ICG‐treated mice. Similarly to what we found in intratumoral injection of CMINs, the increase in miR‐34a level shared a similar response profiles to the increase of NIRF intensity of ICG over experimental time (Figure 6G) and exhibited a good correlation between miR‐34a replacement efficacy and the increase of NIRF intensity in xenograft tumor (Figure 6H), indicating that CMINs‐mediated systemic miR‐34a replacement in tumors could be quantitatively evaluated in a real‐time manner via monitoring the NIRF of ICG. Collectively, these results implied that systemically administration of CMINs could monitor miR‐34a replacement efficacy through activated NIRF pattern.

### 2.7 In Vivo MiR‐34a Replacement Therapy

**Figure 7:**
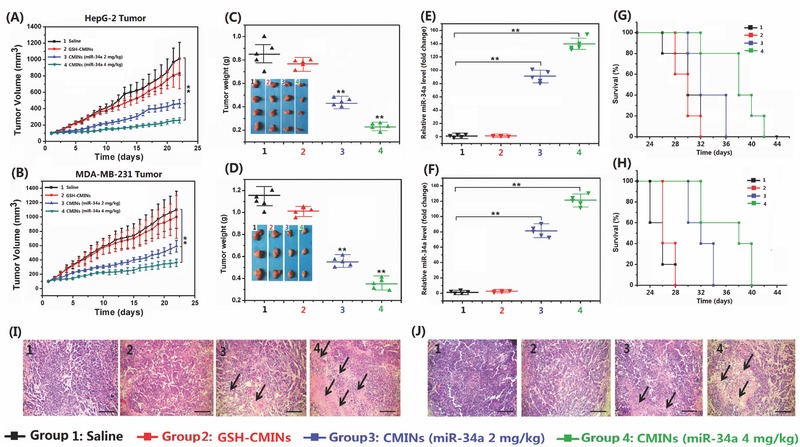
In vivo therapeutic effects of CMINs‐mediated miR‐34a replacement in nude mice A) HepG‐2 and B) MDA‐MB‐231 xenografted nude mice were intravenously administered with saline, GSH‐CMINs, and CMINs with miR‐34a‐equivalent dose of 2 and 4 mg kg^−1^ every 2 d. Tumor volumes were measured and calculated every day. C–D) The mice were sacrificed and the tumors were dissected and weighed at 22 d postinjection. The inset in (C) and (D) showed the representative photos of excised tumors. E,F) Relative miR‐34a expression level in dissected tumors was quantitatively evaluated. G,H) Survival curves of mice after the various treatments. I) The HepG‐2 and J) MDA‐MB‐231 tumor sections were stained with hematoxylin and eosin (H&E) and examined by light microscopy. Tissue paraffin sections were 10 µm thick. The black arrows denote the typical necrotic cells in tumors. Scale bars in all images represent 100 µm. Data represent mean ± SD (*n* = 5, Student's *t*‐test, **p < 0.01).

**Figure 8:**
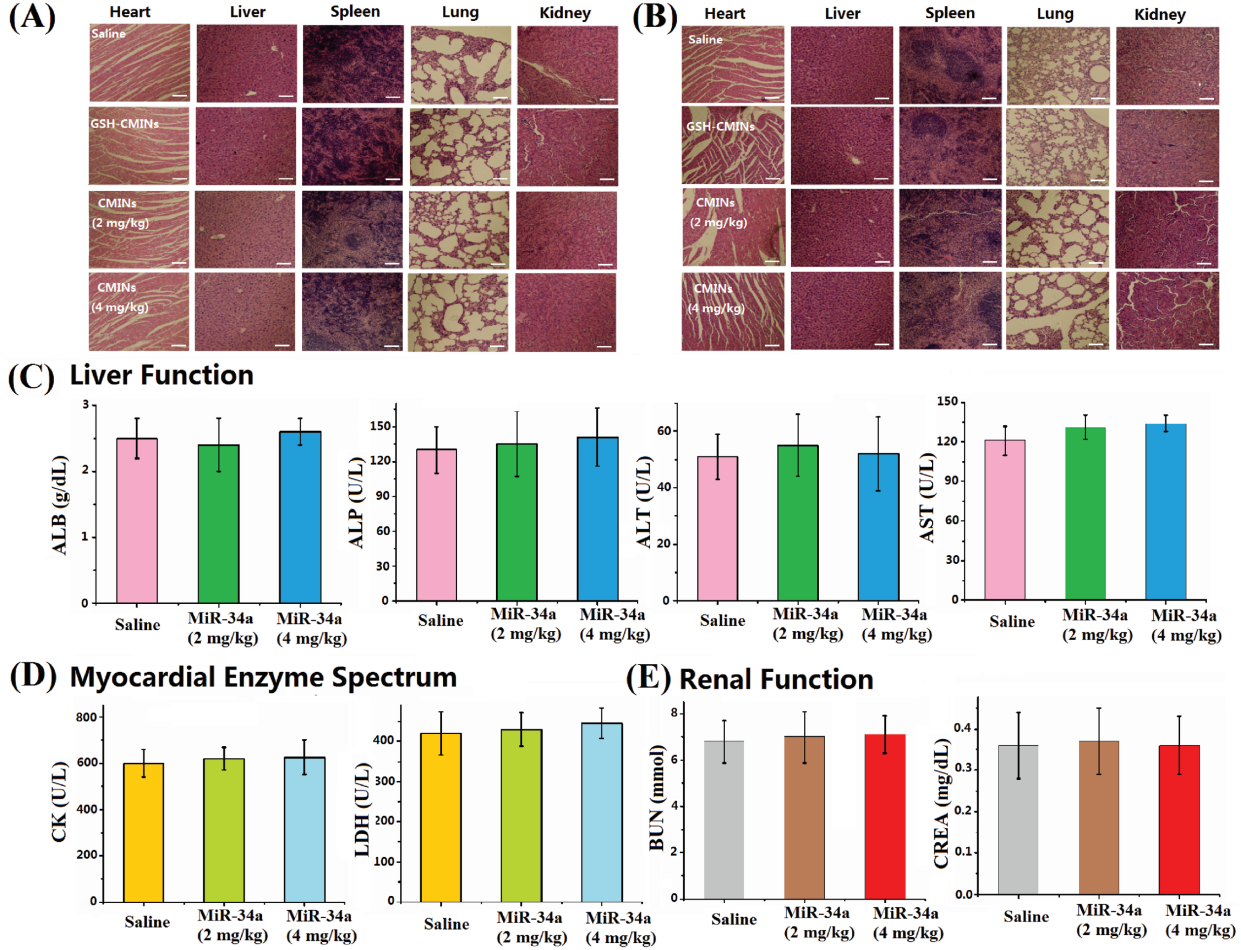
A) HepG‐2 and B) MDA‐MB‐231 tumor bearing mice intravenously injected with saline, GSH‐CMINs, CMINs (2 mg kg^−1^ of miR‐34a), and CMINs (4 mg kg^−1^ of miR‐34a) were sacrificed at 22 d post‐treatment. Representative hematoxylin and eosin (H&E) stained histological images of tissue sections from major organs (heart, liver, spleen, lung, and kidney). Scale bars in all images represent 100 µm. Serum biochemical examination of CMINs‐treated mice. C) Liver function, D) myocardial enzyme spectrum, and E) renal function. ALB, albumin; ALP, alkaline phosphatase; ALT, alanine aminotransferase; AST, aspartate transaminase; CK, creatine kinase; LDH, lactate dehydrogenase; BUN, blood urea nitrogen; CREA, creatinine.

MiR‐34a‐based replacement therapy represents one of the novel strategies for gene therapy against various cancers.^45^ We subsequently established different animal xenograft tumor models of hepatocellular carcinoma and breast cancer to investigate the in vivo antitumor effects of CMINs‐mediated replacement of miR‐34a. The mice were intravenously injected with CMINs with two doses of 2 and 4 mg kg^−1^ of miR‐34a, respectively. The mice received intravenous injection of saline and GSH‐CMINs were used as controls. As shown in **Figure** **7**A,B, compared with the control groups, the treatment with CMINs remarkably inhibited tumor formation of both hepatocellular carcinoma and breast cancer in a dose dependent manner. The tumor weights were also measured and calculated at 22 d postinjection, which are correlated with the above tumor growth data (Figure 7C,D). In addition, the level of miR‐34a was further quantitatively evaluated by qRT‐PCR. The highest miR‐34a level was found in mice treated with CMINs at dose of 4 mg kg^−1^ of miR‐34a, which showed the most potent antitumor efficiency (Figure 7E,F). Moreover, CMINs treatment provided a dramatic survival benefit of mice compared with the control mice (Figure 7G,H). Histological staining showed that CMINs administration could trigger the apoptosis of tumor cells (Figure 7I,J) by suppressing the expression of Bcl‐2 and Notch‐1 in a dose dependent manner (Figure S18, Supporting Information). In addition, no acute in vivo toxicity of CMINs was observed in all animal groups and the body weights of CMINs‐treated mice increased in a manner similar to that of saline group (Figure S19, Supporting Information). To examine any potential damage to major organs after treatments with CMINs, the postmortem histopathology of the heart, liver, spleen, lung, and kidney in all groups was examined. As shown in **Figure** **8**A,B, no obvious morphological differences were observed among all groups. To further assess systemic toxicity in the animals, serum biochemical examination of CMINs‐treated mice was conducted. As shown in Figure 8C–E, all of the parameters associated with the liver function, myocardial enzyme spectrum and renal function in the CMINs‐treated groups appeared to be normal compared with the saline‐injected mice. Taken together, these results suggested that systemically injected CMINs could provide great therapeutic outcomes in variety of tumors with excellent biocompatibility.

## 3 Conclusion

In summary, we have successfully developed NIRF light‐up peptide‐polysaccharide‐inter‐polyelectrolyte nanocomplexes constructed by S‐Arg_4_ peptides and CMD via a self‐assembly approach. The confinement of ICG in the established nanocomplexes led to self‐quenched NIRF through an ACQ mechanism. The dissociation of nanocomplexes in tumor cells can be induced by intracellular GSH and efficiently trigger the release of miR‐34a and ICG, in which the NIRF signal of ICG was reversibly activated upon its release. ICG and miR‐34a may be delivered into tumor tissues by the nanocomplexes though EPR effects and efficiently uptaken by tumor cells. Of particular significance, NIRF intensity of ICG is correlated with the released amount of miR‐34a and miR‐34a replacement efficacy both in vitro and in vivo, either by local or systemic injections of CMINs. In addition, replacement of miR‐34a with CMINs resulted in efficient tumor‐suppressive effects both in vitro and in vivo. The overall data demonstrated that the established nanocomplexes have a great potential to be used as a light‐up theranostic platform with excellent biocompatibility for real‐time monitoring of miR‐34a replacement efficacy and accurate imaging‐guided therapy strategy against tumor.

## 4 Experimental Section

*Materials and Reagent*: CCK‐8 kit and ICG were purchased from Dojindo Molecular Technologies (Tokyo, Japan). 4,6‐diamidino‐2‐phenylindole (DAPI) was purchased from Sangon Biotech (Shanghai, China). S‐Arg_4_ was synthesized by ChinaPeptide Co., LTD (Shanghai, China). Dextran (*M*_w_ ≈ 27 KDa), GSH, BSA and EDC were brought from Sigma‐Aldrich (St. Louis, MO, USA). CMD (degree of substitution of carboxymethylation ≈ 70%) was synthesized according to a previously reported procedure.^1^ All miRNA mimics (with or without cyanine‐3 (Cy‐3) label at 5′ end) used in the human species were synthesized and provided by RiboBio Co. (Guangzhou, China). The sequences of miR‐34a and scramble miRNA are 5′‐UGGCAGUGUCUUAGCUGGUUGU‐3′ and 5′‐UCACAACCUCCUAGAAAGAGUAGA‐3′, respectively. Trypsin‐EDTA, fetal bovine serum (FBS), and penicillin/streptomycin were purchased from Invitrogen (USA). Antibodies were purchased from Abcam, Inc. The PCR primers were synthesized by Sangon Biotech (Shanghai). RNase‐free deionized water was provided by TIANGEN Biotech Co. Ltd (Beijing). The miRNA mimics were dissolving in RNase‐free deionized water. All other chemicals and solvents were of analytical grade commercially available unless specially mentioned otherwise. Ultrapure water (deionized (DI) water) was supplied by a Milli‐Q water system (Millipore, Bedford, MA, USA).

*Preparation of BNs, MINs*, *and CMINs*: BNs were prepared by mixing various mass ratios of S‐Arg_4_ and CMD in aqueous solutions. Briefly, S‐Arg_4_ (2 mg mL^−1^) and CMD (2 mg mL^−1^) were dissolved separately in DI water and filtered through a Millipore 0.22 µm filter prior to experiments. The S‐Arg_4_ solution was added dropwise into 1 mL of CMD solution with different volumes under constant magnetic stirring at room temperature. Subsequently, the mixture was stirred for another 10 min to allow complete formation. Afterward, the resulting BNs were collected by centrifugation (12 000 *g*, 10 min), washed twice with DI water, and dispersed in DI water. The product of BNs was obtained after lyophilization and weighted for analyzing the product yields.

For the preparation of MINs, various amounts of miR‐34a and ICG were first mixed with the CMD solution phase and MINs was formed using the same methods as described above. MINs were obtained after repeated centrifugation, washing in DI water for three times and finally dispersed in 1 mL of DI water for further use.

Cross‐linked MINs (CMINs) were obtained by adding 2 mg of EDC to the obtained MINs solution and incubated overnight. After 12 h reaction, the resultant CMINs were obtained by repeated washing and centrifugation (10 000 *g*, 10 min) for three times in DI water to remove residual reagents and finally dispersed in 1 mL of DI water for further use.

*Characterization*: The DLS measurements were performed on a Malvern Zetasizer nano ZS instrument (Malvern Instruments, UK) to determine the size and zeta potential. The degradation kinetics of CMINs was quantified using DLS measurements. The degree of dissociation can be described as *I*/*I*_0_ × 100%, where *I*_0_ is the initial SLI of CMINs and *I* is the SLI measured at the indicated time interval. The morphologies of nanocomplexes were determined by TEM (Tecnai G2 20 S‐TWIN, FEI Company, Philips, the Netherlands) after negative staining with 3% uranyl acetate solution. FT‐IR spectra were collected on a Spectrum One spectrometer (Perkin Elmer, USA). The absorption spectra of ICG and CMINs were recorded by UV–vis spectrometer (UV‐2501 PC, Shimadzu, Japan). Fluorescence spectra were obtained by LS‐55 Fluorescence Spectrometer (PerkinElmer, Fremont, CA) with excitation at 735 nm to monitor ICG fluorescence. Fluorescence images were acquired with the ex/in vivo imaging system (CRi, Woburn, MA). To qualitatively evaluate the EE and LC of ICG and miR‐34a in MINs, Cy‐3‐labeled miR‐34a was used as an indicator. The EE and LC of Cy‐3‐miR‐34a and ICG were measured by measuring the fluorescence intensity of ICG and Cy‐3 in the supernatant after preparation of MINs.

*Stability Test and Protein Adsorbtion Assays*: CMINs were suspended in DI water, PBS of pH 7.4 and 150 × 10^−3^ m sodium chloride (NaCl) solution, respectively. The particle sizes were determined at 0, 1, 3, and 7 d by DLS as described above. For the heparin stability test, MINs and CMINs were incubated with 5 and 10 mg kg^−1^ of heparin for 30 min at room temperature. Agarose gel electrophoresis was then carried out on 2% agarose gel and imaged by a UV gel image.

BSA was used as a model protein to determine the protein adsorption of CMINs. CMINs (1 mg mL^−1^) were incubated with PBS (pH 7.4) containing 1 mg mL^−1^ of BSA. After incubation at 37 °C for 2 h, 200 µL of each sample were withdrawn after vortex to ensure homogeneity and centrifuged (8000 *g*, 10 min). The protein concentration in the supernatant was quantified using UV–vis spectroscopy. Then, the adsorbed proteins were calculated against a standard calibration curve of the proteins.

*Electrophoretic Gel Assay*: Embedding of miR‐34a in the CMINs was monitored by a gel electrophoresis. The gels were prepared with 2% agarose in tris‐acetate‐ethylenediaminetetraacetic acid (EDTA) buffer containing 0.5 µg mL^−1^ GelREDTM (Biotium, USA). For gel retardation assay, samples were incubated at room temperature for 15 min, 10% glycerine was then added to each sample. Gel electrophoresis was carried out at 110 V for 10 min and the gel was subsequently photographed using Alpha Innotech gel imager system.

Serum degradation assays were conducted as below: free miR‐34a and the CMINs solutions were incubated with 50% serum. The mixed solutions were then incubated at 37 °C for extended time points. 15 µL of the mixtures were taken out at the indicated time interval and then mixed with 5 µL 2% sodium dodecyl sulfonate (SDS) and 2.5 µL 10% glycerine. Then, the above mixtures were loaded onto 2% argarose gel and gel electrophoresis was carried as described above.

*In Vitro MiR‐34a and ICG Release Profiles*: 2 mL of CMINs (0.74 mg mL^−1^) were put into two separate dialysis bag (molecular weight cut‐off (MWCO) 3500 Da for ICG and MWCO 50 000 Da for Cy‐3‐miR‐34a) and then the dialysis bags were immersed in 10 mL of RNase‐free PBS of pH 7.4 with 2 × 10^−6^ m and 10 × 10^−3^ m GSH at 37 °C with constant shaking at 100 rpm, respectively. At the indicated time interval, 0.5 mL of medium was taken out from the solution and 0.5 mL of fresh PBS was added again after each sampling. The released ICG were measured by UV–vis–NIR spectrophotometer. The released Cy‐3‐miR‐34a was detected by measuring the fluorescence intensity of Cy‐3 (λ_ex_ 550 nm and λ_em_ 570 nm). The release of Cy‐3‐miR‐34a from CMINs in the presence of 10 × 10^−3^ m GSH was also evaluated by agarose gel electrophoresis. CMINs were incubated in the presence of 10 × 10^−3^ m GSH for different time intervals (1, 5, 10, 30, 60, 120, and 180 min), then loaded on a 2% agarose gel as described above.

*Cell‐Lines, Culture and Measurement of Cytotoxicity*: Human hepatocellular carcinoma HepG‐2 cell line and human breast cancer MDA‐MB‐231 cell line were originally purchased from American Type Culture Collection. The cells were cultured under standard conditions in DMEM medium supplemented with 10% (v/v) FBS, 100 units mL^−1^ penicillin, and 100 µg mL^−1^ streptomycin at 37 °C with 5% CO_2_ in a humidified chamber. For cell cytotoxicity assay, HepG‐2 cells were seeded in 96‐well plates at a density of 1 × 10^4^ cells per well and incubated at 37 °C incubator with 5% CO_2_. After incubation overnight, the cells were treated with various concentrations of Scr‐CMINs. After 48 h incubation, cell viability was evaluated by CCK‐8 assay.

*Cellular Uptake and Fluorescence Imaging Capability of CMINs*: 1 × 10^5^ HepG‐2 cells were cultured in a 20 mm glass‐bottom dish over night. HepG‐2 cells were incubated with CMINs (3.2 µg of miR‐34a and 3.0 µg of ICG) for 0.5, 1, 2, and 4 h, respectively. The cells were thereafter washed with PBS and stained by Hoechst‐33258. The images were captured under a Zeiss LSM780 confocal microscopy (Zeiss Cp., Germany). Flow cytometry analysis was conducted to quantitatively analyze the cellular uptake efficacy of CMINs. HepG‐2 cells (5 × 10^4^) were seeded in 24‐well plates and cultured to ≈50% confluence. The cells were incubated at 37 °C with CMINs for different time points. The cells were then trypsinized, washed with PBS, and subjected to analysis using a BD FACSCalibur flow cytometer (BD Bioscience, Bedford, MA). The results were processed using FlowJo software.

*In Vitro Proliferation, Wound Healing, Transwell, and Apoptosis Assay*: 1 × 10^3^ HepG‐2 cells were seeded in a 96‐well plate overnight. 100 × 10^−9^ m concentration of miR‐34a in CMINs were added to each well. Saline‐ and Scr‐CMINs‐treated cells were used as negative control. Lipofectamine encapsulated miR‐34a (lipo/miR‐34a)‐treated cells were used as positive control. Lipo/miR‐34a complexes were prepared according to the manufacture's protocol. CCK‐8 assay was performed to determine the cell viability after incubation for extended time points. Each experiment was performed in triplicate.

A wound healing assay was performed according to the previous described procedure. Briefly, HepG‐2 cells (1 × 10^5^) were seeded in 6‐well plates for 24 h. When the cell confluence reached 80%, the cells were treated with various formulations for 4 h and then scratched with a 1 mL sterile pipet tip. The medium was replaced with fresh medium containing 20% FBS and the cells were incubated for additional 48 h. Finally, scratched areas were visualized under a light microscope to evaluate the migration rate of cells into the scratched area. At the endpoint of wound healing assay, the total cell number was also normalized by CCK‐8 assay.

Transwell migration assay was carried out in a 24‐well using matrigel invasion chambers with 8 µm pores according to the manufacturer's instructions (Corning). HepG‐2 cells (1 × 10^5^) were placed in the upper chamber in serum‐free medium. Medium containing 20% FBS in the lower chamber served as a chemo‐attractant. After incubation of saline, Scr‐CMINs, CMINs (100 × 10^−9^ m miR‐34a), and lipo/miR‐34a (100 × 10^−9^ m miR‐34a) for 48 h, the upper surface of the membrane was gently scraped to remove nonmigrating cells and then washed twice with PBS. The membrane was fixed in 4% paraformaldehyde for 10 min and stained with 0.2% crystal violet for 10 min. The migrating cells were calculated by counting five random fields per filter under a light microscope. At the end of transwell assay, we also normalized with the total cell number at the lower chamber by CCK‐8 assay.

Apoptosis was measured by using Annexin V‐fluorescein isothiocyanate (FITC)/(propidium iodide) PI staining according to the apoptotic assay kit. After treatment with saline, Scr‐CMINs, CMINs (100 × 10^−9^ m miR‐34a) and lipo/miR‐34a (100 × 10^−9^ m miR‐34a) for 48 h, HepG‐2 cells were divested, washed, and processed by using Annexin/PI apoptosis detection kit. The stained cells were then subjected to flow cytometric analysis (FACSCalibur, BD, USA) after suspended in PBS.

*Determination of Gene and Protein Expression by qRT‐PCR and Western‐Blot Analysis*: qRT‐PCR study was performed to analyze miR‐34a level and their downstream target mRNA levels according to the previous methods. Total RNA was isolated using Trizol reagent according to the manufacturer's instruction (Invitrogen, USA). U6 was used as an endogenous control. The sequences of the primers are

MiR‐34a: 5′‐AACAACCAGCTAAGACACTGCCA‐3′;
U6: 5′‐ GCTTCGGCAGCACATATACTAAAAT ‐3′

To analyze the protein levels of Ran and Δp63, a Western‐blot study was performed as described previously. GAPDH was used as an internal protein (Cell signaling, USA).

*Animals*: Healthy female BALB/c nude mice (18–20 g, four to six weeks old) were provided by the Beijing Vital River Laboratories (Beijing, China). Animal care and handing procedures were in accordance with the Experimental Ethics Committee in Beijing. All mice were housed (a group of five) in a clean environment supplemented with enough water and fresh food and were under a 12 h light/dark environment. The mice were acclimatized for 7 d prior to the in vivo experiments.

*Hemolysis Assay*: The blood compatibilities of CMINs were evaluated by hemolysis test on mice red blood cells (RBCs). The RBCs were separated according to the previous literature. Briefly, the anticoagulated blood was diluted with PBS and centrifuged at 1500 rpm for 10 min under 4 °C. The erythrocyte pellets at the bottom of centrifuge tube were collected and washed three times with 1 × PBS. The resultant RBCs suspension was diluted with PBS, producing a stock RBCs solution. RBCs upon incubation with water and 1 × PBS were used as positive and negative controls, respectively. A total of 0.5 mL of RBCs stock solution was incubated with different concentrations of CMINs, the volumes of the mixtures were adjusted to 1.0 mL with 1 × PBS. The resultant mixtures were incubated at 37 °C for 4 h and centrifuged at 1500 rpm for 10 min. The absorbance of the supernatant solution of test (Atest), positive control (Apos) and negative control (Aneg) at 540 nm were measured using a UV–vis spectrophotometer. Each set of experiments was carried out for three times. The hemolysis (%) = [(Atest – Aneg)/(Apos − Aneg)] × 100%.

*In Vivo Biodistribution and Monitoring Investigation by NIRF Bioimaging and qRT‐PCR Method*: HepG‐2 cells (5 × 10^6^) were injected on each flank of mice to establish the two tumor‐bearing mice (*n* = 8 mice per group). When the tumors reached about 100 mm^3^, CMINs (100 µL, 3 µg of ICG) and free ICG (100 µL, 3 µg) were intratumorly injected at the left and right tumor, respectively. At 10 min, 30 min, 60 min, 3 h, 6 h, 12 h, and 24 h after injection, mice were anesthetized and imaged using the ex/in vivo imaging system (CRi, Woburn, MA). At the indicated time intervals, the NIRF intensity from each tumor was analyzed by the instrument software. Meanwhile, the tumors were immediately excised, homogenized, lysed, and then measured by qRT‐PCR to quantitatively determine the miR‐34a level.

In vivo whole‐body NIRF imaging assay was conducted to evaluate the biodistribution and potential tumor imaging ability after systemic injection of CMINs. HepG‐2 tumors were established by injected with 5 × 10^6^ cells in the right flank of female balb/c mice. When the tumor size grew to around 100 mm^3^, the mice were administered via tail injection of 100 µL of saline, ICG solution (10 µg), CMINs (100 µL, 10 µg of ICG), and CMINs (100 µL, 10 µg of ICG) pretreated with 10 × 10^−3^ m GSH (GSH‐CMINs) for 2 h. At 1, 6, 12, 24, and 48 h postadministration, mice were anesthetized and imaged as described above. After 48 h, the mice after in vivo imaging were sacrificed and the tumors, hearts, livers, spleens, lungs, kidneys were collected for imaging. Microscopy imaging of frozen tumor slides were performed as following: After 48 h postinjection, animals were sacrificed, and tumors were collected. Then, the tumor tissues were frozen and cut into 10 µm thickness using a Leica cryostat. After that, the slides were then imaged by fluorescence microscope. In additional experiments, at 1, 6, 12, 24, and 48 h postadministration of saline, CMINs, and GSH‐CMINs, the tumors were NIRF imaged and analyzed. The mice were sacrificed and the tumors were immediately excised, homogenized, lysed, and then measured by qRT‐PCR to quantitatively determine the miR‐34a level.

*In Vivo Evaluation of MiR‐34a Replacement Therapy Effect*: HepG‐2 and MDA‐MB‐231 tumors were established by injected with 5 × 10^6^ cells in the right flank of female balb/c mice. When the tumor size grew to around 100 mm^3^, the mice were randomly divided into five groups (5/group). The tumor‐bearing mice were administrated by intratail‐vein injection with saline, CMINs at a miR‐34a (equivalent dose of 2 and 4 mg kg^−1^) and GSH‐CMINs every 2 d for two weeks. The injection was repeated for five times at an interval of 2 d. The mice were weighed daily by electronic balance and the tumor sizes were measured by vernier calipers for two weeks. The tumor volume was calculated by the following formula: Tumor volume = (*L* × *W* ^2^)/2. To quantify the miR‐34a level and its target gene expression, the tumors were immediately homogenized, lysed, and then measured by qRT‐PCR measurements at the end of the experiment. At last, the tumor tissues and major organs were fixed in 10% formalin and used for hematoxylin and eosin staining by the Peking University Health Science Center. The histological sections were observed under optical microscopy. The survival rate of mice treated with various treatments were also recoded and analyzed. For each group subjected to the corresponding treatment, the survival rate was calculated by dividing the number of surviving mice at different days of post‐treatment with the total number of mice before treatment.

*Ex Vivo Hemotology Analysis*: Serum biochemical analysis was performed to systemically evaluate the biosafety of CMINs after systemic administration. After intravenously injected with saline and CMINs (2 and 4 mg kg^−1^), mice (*n* = 5) were sacrificed and sera were collected for further blood biochemical studies (Charles River Laboratories, Beijing, China).

*Statistical Analysis*: All data are determined as mean ± SD and all experiments were performed at three times. Statistical significance of the data was considered by the one‐way analysis of variance (ANOVA). Values of *p < 0.05 and **p < 0.01 were considered statistically significant.

## Conflict of Interest

The authors declare no conflict of interest.

## Supporting information

Supplementary
