# Supplementary material for: In Situ Monitoring of MicroRNA Replacement Efficacy and Accurate Imaging‐Guided Cancer Therapy through Light‐Up Inter‐Polyelectrolyte Nanocomplexes
Source: Adv Sci (Weinh). 2018 Jan 19;5(4):1700542. doi: 10.1002/advs.201700542 (PMC5908377; doi:10.1002/advs.201700542)
Supplement: Supplementary file 1 — Supplementary [file ADVS-5-1700542-s001.pdf]

Copyright WILEY-VCH Verlag GmbH & Co. KGaA, 69469 Weinheim, Germany, 2018.

ADVANCED SCIENCE
Open Access

Supporting Information

for *Adv. Sci.*, DOI: 10.1002/advs.201700542

In Situ Monitoring of MicroRNA Replacement Efficacy and Accurate Imaging-Guided Cancer Therapy through Light-Up Inter-Polyelectrolyte Nanocomplexes

Xiongwei Deng, Zhaoxia Yin, Jianqing Lu, Xianlei Li, Leihou Shao, Caiyan Zhao, Yishu Yang, Qin Hu, Yan Wu,* and Wang Sheng**

Copyright WILEY-VCH Verlag GmbH & Co. KGaA, 69469 Weinheim, Germany, 2016.

Supporting Information

In Situ Monitoring of MicroRNA Replacement and Accurate Imaging-Guided Cancer Therapy through Light-Up Inter-Polyelectrolyte Nanocomplexes

Xiongwei Deng, Zhaoxia Yin, Jianqing Lu, Xianlei Li, Leihou Shao, Caiyan Zhao, Yishu Yang, Qin Hu, * *Yan Wu,* **and Wang Sheng* *

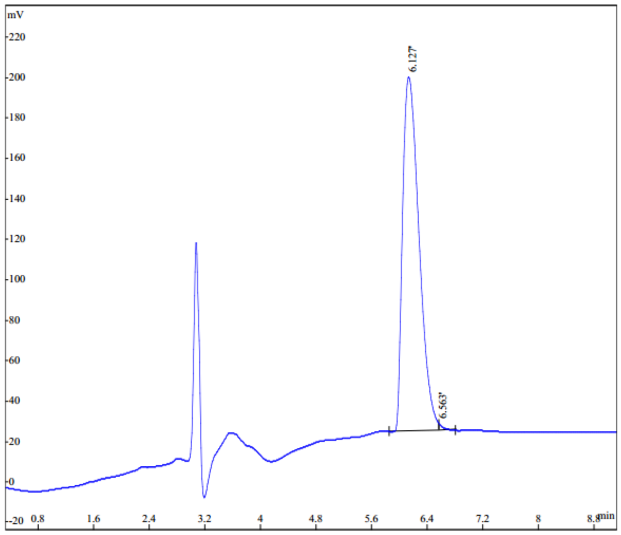

Rank	Time	Name	Conc.	Area
1	6.127		99.5	2884012
2	6.563		0.4982	14439
Total			100	2898451

Figure S1. High performance liquid chromatography (HPLC) analysis of purified S-Arg_4 peptides with reverse phase C18 column.

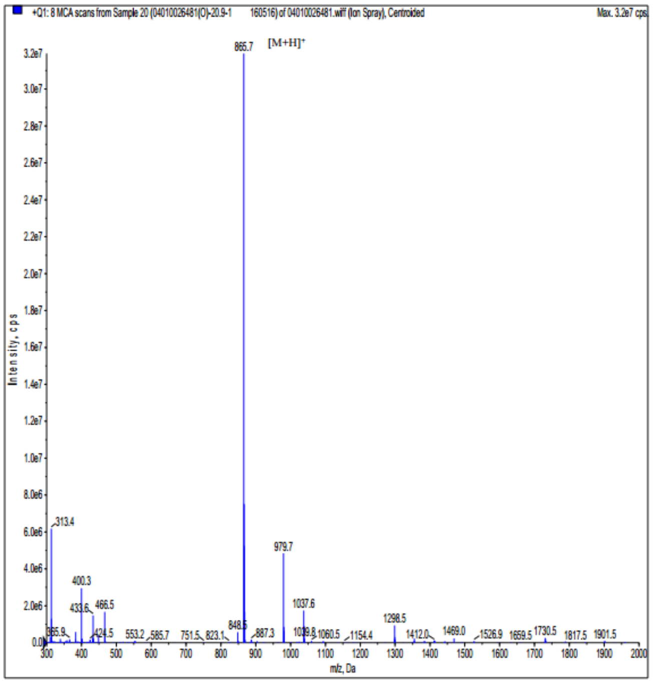

Figure S2. Liquid chromatography-mass spectrometry (LC-MS) analysis of S-Arg_4 peptides prepared by solid phase peptide synthesis. The major peak (MW=865.7) is consistent with the estimated product molecular weight of 865.06.

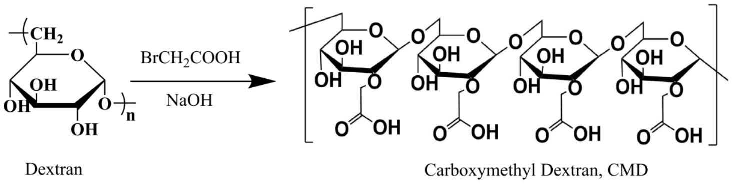

Scheme S1. Synthesis process of carboxymethyl dextran (CMD).

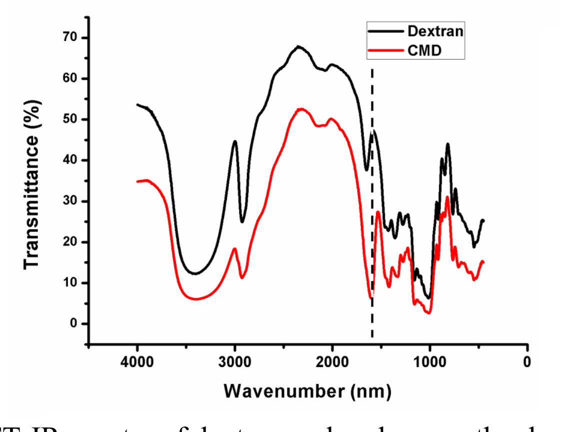

Figure S3. FT-IR spectra of dextran and carboxymethy dextran (CMD).

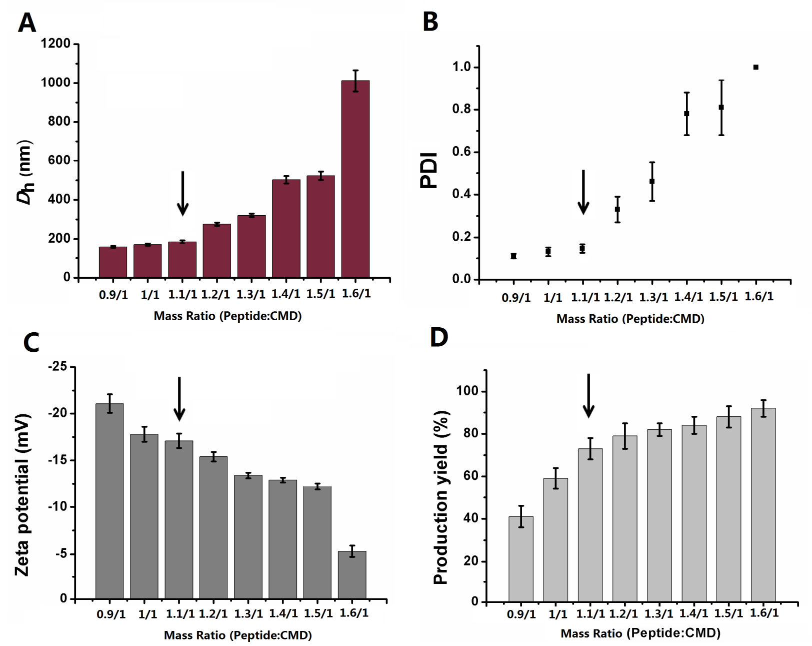

Figure S4. Optimization and characterization of blank nanocomplexes (BNs) composed of various mass ratios of S-Arg$_4$ and CMD. (A) Hydrodynamic size, (B) polydispersity index (PDI) and (C) surface zeta potential determined by DLS analysis. (D) Production yield of the BNs.

Table S1. Effects of mass ratio of materials on embedding efficiencies (EE) and loading content (LC) of miR-34a and ICG

S-Arg$_4$:CMD:MiR-34a:ICG(mass ratio)	1.1:1: 0.01:0.01	1.1:1: 0.025:0.025	1.1:1: 0.05:0.05	1.1:1: 0.075:0.075	1.1:1: 0.1:0.1
Embedding efficiency of miR-34a (EE) %	93.1	87.2	75.2	51.2	41.1
Loading content of miR-34a (LC) %	0.66	1.41	2.55	2.60	2.79
Embedding efficiency of ICG (EE) %	90.3	82.5	70.2	45.2	38.3
Loading content of miR-34a (LC) %	0.63	1.40	2.45	2.30	2.61

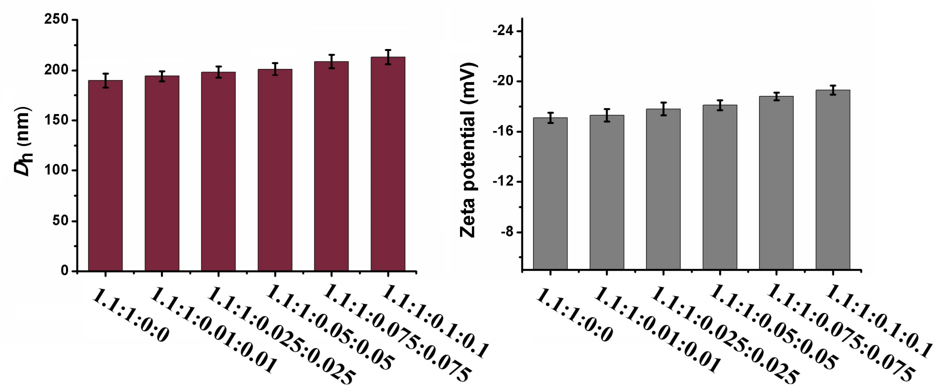

Figure S5. Average sizes and zeta potentials of MINs upon embedding with different amounts of miR-34a and ICG.

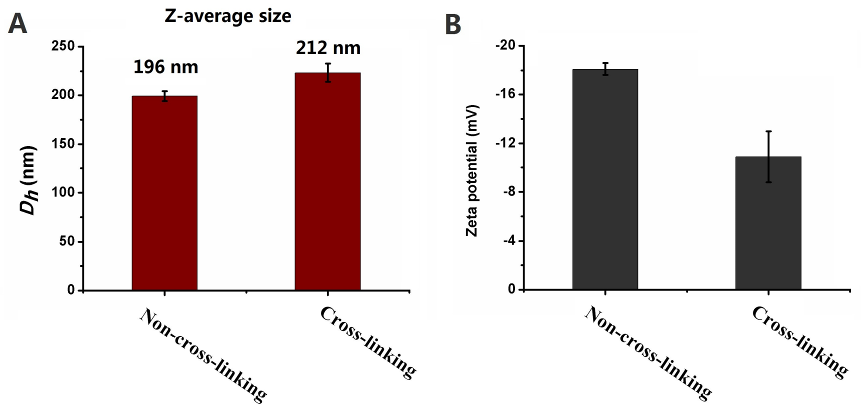

Figure S6. Size and zeta potential of uncross-linked MINs and cross-linked CMINs determined by DLS measurements.

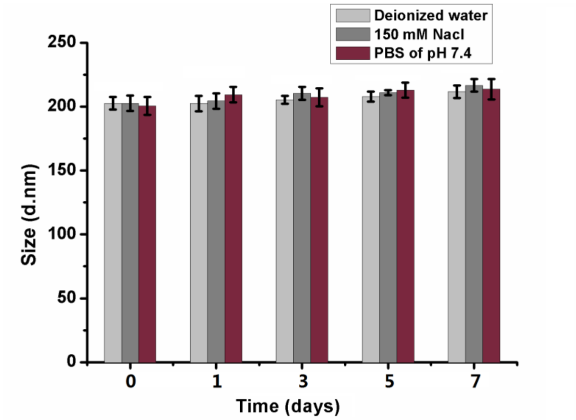

Figure S7. Post-formulation colloidal stability of CMINs dispersed in different solvent environments at 0, 1, 3, 5 and 7 days based on the average hydrodynamic diameters.

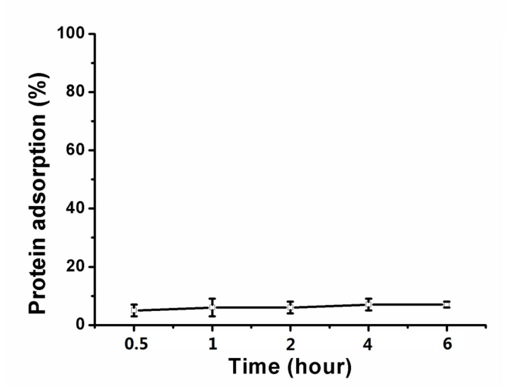

Figure S8. Bovine serum albumin (BSA) adsorption assays on the CMINs after different time co-incubation at 37 °C.

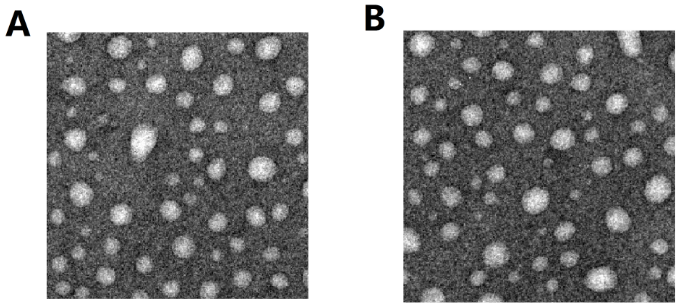

Figure S9. TEM images of CMINs in PBS of pH 7.4 in the presence of 2 μM GSH for 2 h (A) and 4 h (B).

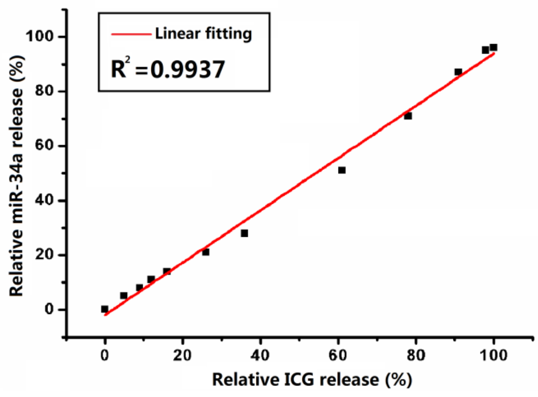

Figure S10. Linear correlation between the ratios of ICG release and miR-34a release.

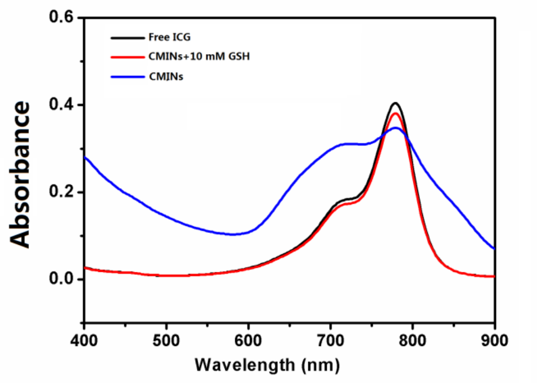

Figure S11. UV/Vis spectra of free ICG solution, CMINs and CMINs incubated with 10 mM GSH for 4 h.

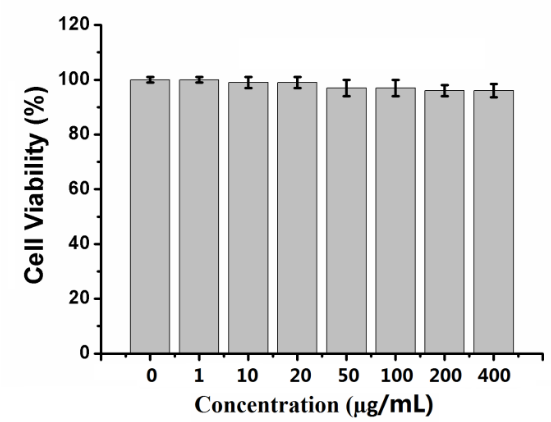

Figure S12. Cell cytotoxicity. HepG-2 incubated with different concentrations of scramble miRNA-encapsulating CMINs (Scr-CMINs). Error bars represent standard deviations of three dependent experiments.

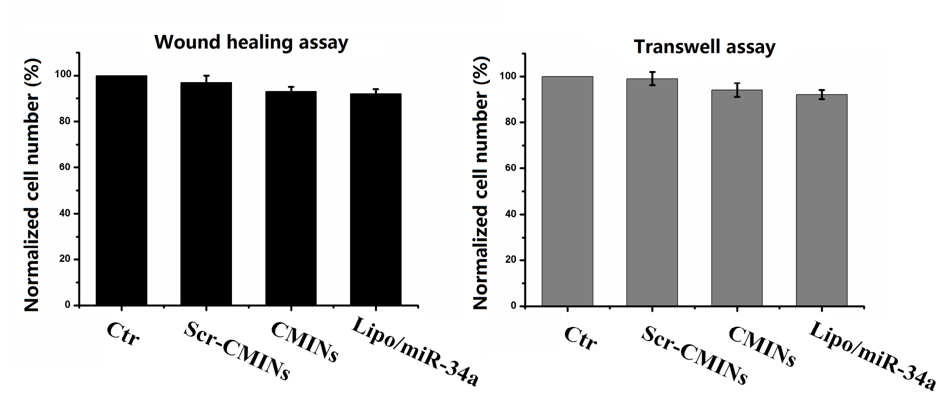

Figure S13. Normalized cell number at the endpoint of wound healing and transwell assays by CCK-8 assays.

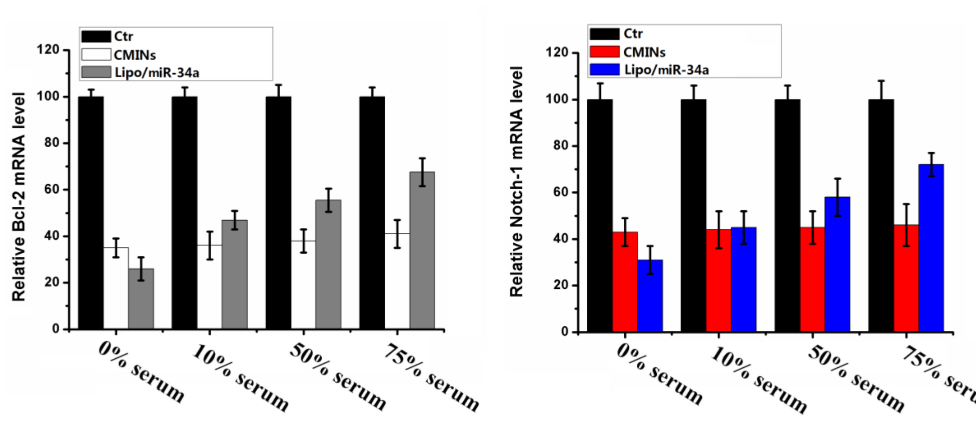

Figure S14. The expression of Bcl-2 and Notch-1 was quantitatively detected by qRT-PCR upon the treatment of lipo/miR-34a and CMINs in the presence of different concentrations of serum.

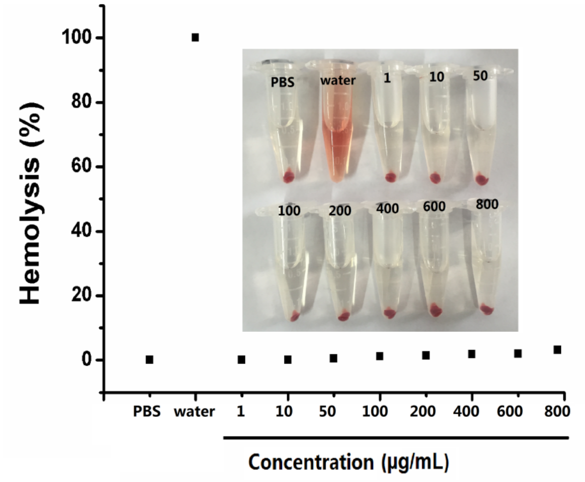

Figure S15. Hemolysis assay with different concentrations of CMINs (negative control: PBS; positive control: water).

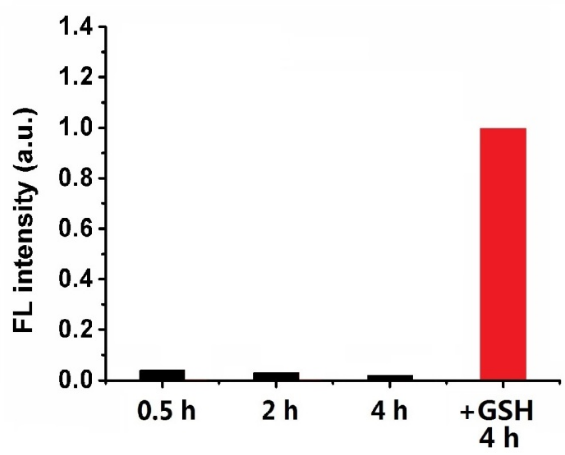

Figure S16. Fluorescence Images and relative fluorescence intensity of CMINs (100 µL, 0.2 µg of ICG) at 0.5 h, 2 h and 4 h in mice blood with or without 10 mM GSH.

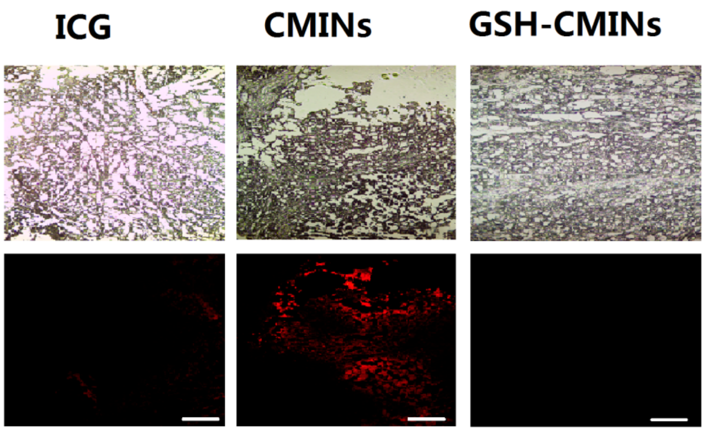

Figure S17. Histological and fluorescent images of frozen tumor sections (10 μm thick). ICG fluorescence is shown in red. Scale bars in all images in are 100 μm.

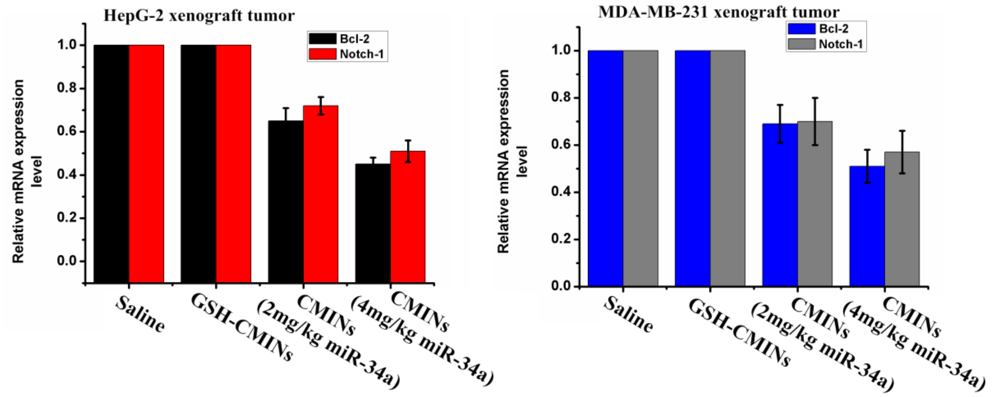

Figure S18. QRT-PCR was used to measure the Bcl-2 and Notch-1 expression at transcriptional level in tumors upon treatment of CMINs with different doses of miR-34a.

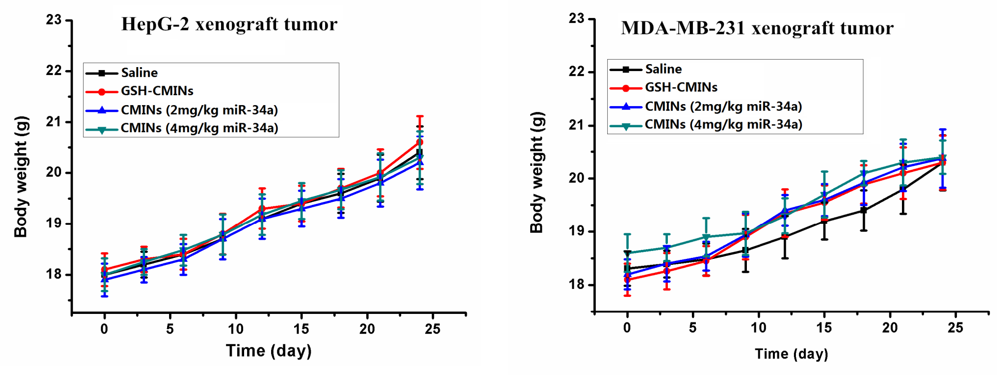

Figure S19. Body weights measurements. No significant differences in body weights were found in two tumor mouse models upon treatment with saline, GSH-CMINs, CMINs (2 mg/kg of miR-34a) and CMINs (4 mg/kg of miR-34a).
